# Outpouching in the Esophagus: An Uncommon Endoscopic Finding of Esophageal Intramural Pseudodiverticulosis in the Absence of Esophageal Candidiasis

**DOI:** 10.7759/cureus.39805

**Published:** 2023-05-31

**Authors:** Daniyal Raza, Maryam Mubashir, Hassaan A Zia

**Affiliations:** 1 Internal Medicine, Louisiana State University (LSU) Health Shreveport, Shreveport, USA; 2 Gastroenterology and Hepatology, Louisiana State University (LSU) Health Shreveport, Shreveport, USA

**Keywords:** esophageal adenocarcinoma, aids, hiv, pseudodiverticulosis, esophageal stricture

## Abstract

We present a case of a 51-year-old female with a history of acquired immunodeficiency syndrome (AIDS) and medication non-compliance who experienced progressively worsening dysphagia to both solids and liquids over a three-month period. The patient underwent an esophagogastroduodenoscopy (EGD), which revealed multiple small pseudodiverticula without any other notable abnormalities. Subsequently, a barium esophagogram was performed, confirming the presence of multiple esophageal pseudodiverticula. Biopsies taken during the procedure showed chronic inflammatory changes, with no evidence of viral or fungal elements. In light of the patient's HIV history and the absence of esophageal candidiasis, the diagnosis of esophageal intramural pseudodiverticulosis (EIP) was made. The patient was initiated on highly active antiretroviral therapy (HAART) and received high-dose proton pump inhibitors (PPIs). Remarkably, the patient reported a complete resolution of her dysphagia symptoms during the follow-up visit. Risk factors associated with EIP include HIV infection, diabetes mellitus (DM), and esophageal candidiasis. To confirm the diagnosis, a barium esophagogram is considered the preferred imaging study. The management of EIP focuses on PPI therapy, the dilation of strictures if present, and addressing the underlying etiology. Given the association between EIP and esophageal malignancies, surveillance endoscopy may be recommended in these patients. This case highlights the importance of considering EIP as a potential cause of dysphagia, particularly in individuals with HIV/AIDS, even in the absence of esophageal candidiasis. Prompt diagnosis and appropriate management can lead to symptom resolution and improved quality of life for affected patients.

## Introduction

Esophageal intramural pseudodiverticulosis (EIP) is a relatively uncommon benign condition characterized by multiple flask-shaped outpouchings of the esophageal wall. It presents with symptoms such as dysphagia; odynophagia; and, in rare cases, upper gastrointestinal bleeding [1]. The outpouchings correspond to the ducts of the submucosal glands in the esophagus, giving rise to the term EIP [2]. The first documented case of esophageal intramural pseudodiverticulosis (EIP) was reported in 1960 by Mendl et al. The case involved a 56-year-old miner who had a history of consuming excessive amounts of alcohol and cigarettes [3]. Since then, only around 200 cases have been reported in the medical literature. According to a study that examined 14,350 barium swallow esophagrams, the clinical condition of esophageal intramural pseudodiverticulosis (EIP) was identified in only 21 out of the total patient population [4]. The etiology of EIP is unknown; however, multiple studies have attributed alcohol consumption and cigarette smoking as leading risk factors [5]. Candidiasis has been observed to have an association with esophageal pseudodiverticula in certain cases. However, the exact nature of this relationship is not fully understood [6]. Esophageal intramural pseudodiverticulosis (EIP) in individuals with HIV/immunodeficiency syndrome (AIDS) is considered rare, particularly in the absence of esophageal candidiasis. We present a unique case of dysphagia in a patient with AIDS who was diagnosed with EIP, despite the absence of concurrent esophageal candidiasis.

## Case presentation

**Figure 1:**
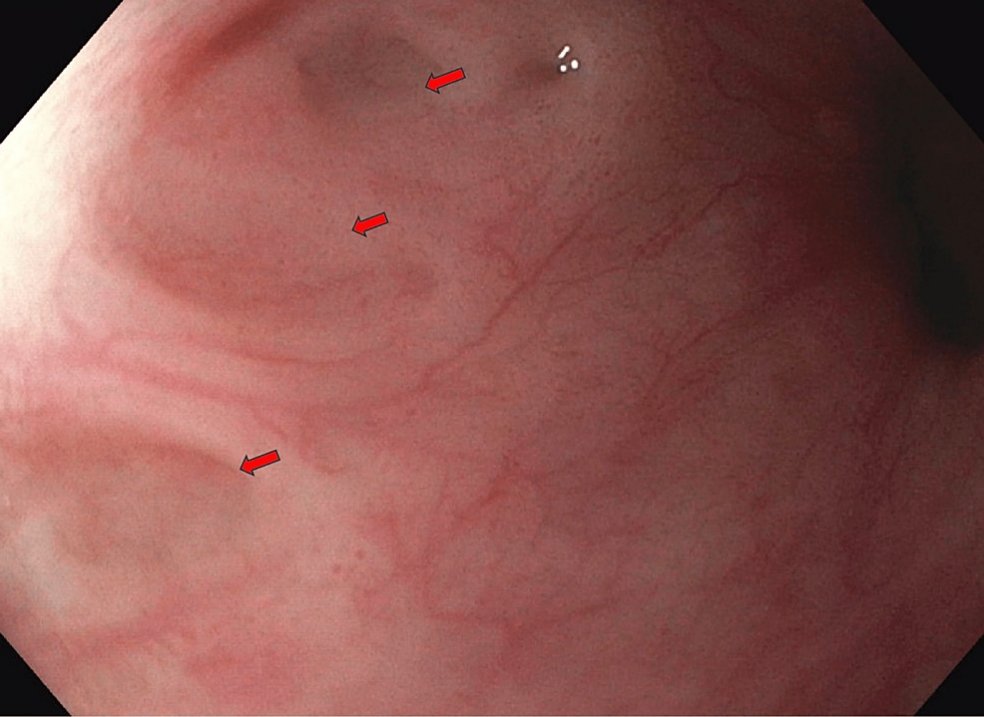
The red arrows show pseudodiverticula

**Figure 2:**
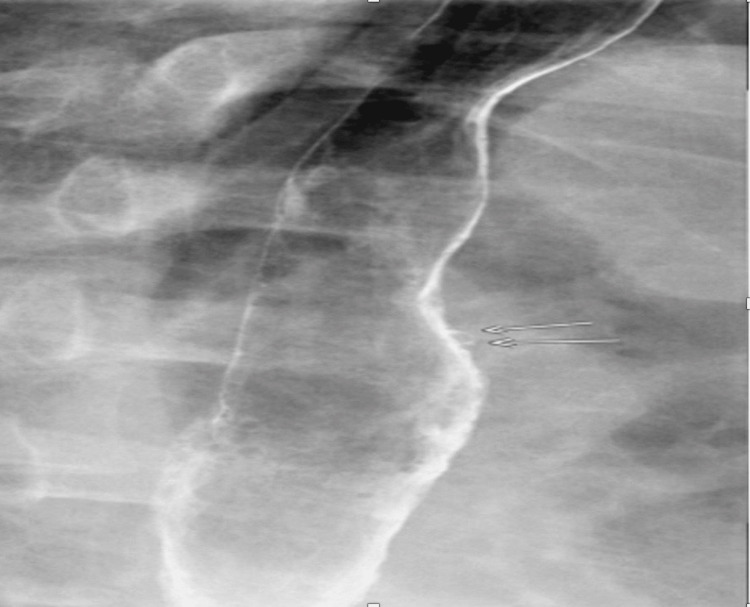
Barium esophagogram showing pseudodiverticula

A 51-year-old female with a past medical history of HIV/AIDS, syphilis, and uveitis presented with a three-month history of progressively worsening solid and liquid food dysphagia, odynophagia, and retrosternal burning. These symptoms resulted in decreased oral intake and subsequent weight loss. She denied experiencing nausea, vomiting, abdominal pain, constipation, melena, hematochezia, hematemesis, or other active concerns or having a history of smoking. She reported that she had been unable to afford her highly active antiretroviral therapy (HAART) for HIV due to financial constraints. In addition, she stated that she was not currently taking any other medications. During the examination, she had a low body mass index (BMI) of 18.0 and oral thrush, but the rest of the examination was unremarkable. Her blood counts and chemistries did not reveal any significant abnormalities. She had a positive HIV viral load with a cluster of differentiation 4 (CD4) count of 0.5 cells/mm^3^. To evaluate the dysphagia, an upper endoscopy was performed followed by a barium esophagogram. The lower third of the esophagus demonstrated multiple diminutive pseudodiverticula, with the rest of the examination being insignificant for any pathology (Figures 1-2). The histopathology analysis of esophageal biopsies indicated chronic inflammatory changes without viral or fungal elements. HAART was restarted, and a high dose of proton pump inhibitor (PPI) was initiated. During the three-month follow-up visit, the patient reported a complete resolution of symptoms. Given the absence of dysphagia concerns, a repeat esophagogastroduodenoscopy (EGD) was not performed during the follow-up.

## Discussion

The pathogenesis of esophageal intramural pseudodiverticulosis (EIP) involves the pathological dilation of excretory ducts of the submucosal glands, leading to the formation of pseudodiverticula and submucosal fibrosis [7]. The exact cause of EIP is unknown. Several comorbidities and conditions have been associated with an increased risk of developing EIP, including Crohn's disease, diabetes mellitus (DM), chronic alcohol abuse, esophageal candidiasis, esophageal malignancies, Mallory-Weiss syndrome, HIV infection, gastroesophageal reflux disease (GERD), and corrosive esophageal injury. The disorders of esophageal motility, such as achalasia and hypoperistalsis, have also been linked to EIP [8-10]. Approximately 50% of the patients with EIP may have concomitant esophageal candidiasis, but it remains unclear whether esophageal candidiasis is the cause or consequence of EIP. In the presented case, the patient had oral candidiasis and HIV infection with a low CD4 count due to medication non-compliance. This scenario raised suspicion of esophageal candidiasis as a preliminary cause for dysphagia; however, the endoscopic examination and biopsies showed no viral or fungal element. It is noted that the patient did not have a history of prior esophagogastroduodenoscopy (EGD) as there was no previous indication for it based on her medical history. The initial presentation of EIP is variable, with some presenting acutely with food impaction, whereas others have long-standing dysphagia to either solids, liquids, or both.

Dysphagia is the most common clinical manifestation of EIP, and a barium esophagogram is considered the gold standard for diagnosing EIP, as it can demonstrate multiple flask-shaped diverticula [11]. An upper endoscopy can be performed to corroborate imaging findings and to evaluate any infectious and structural etiologies [12,13]. Esophageal strictures can be present in up to 90% of the patients. About 50% of the patients have stenosis of the esophagus at the time of the presentation. Hence, the patients presenting with proximal esophageal stenosis should have a high index for suspicion of EIP [14]. As the underlying pathophysiology of EIP is poorly understood, there is currently no standard treatment. Management focuses on symptomatic relief and addressing predisposing risk factors. This may involve the use of highly active antiretroviral therapy (HAART), antifungal medications, proton pump inhibitors (PPIs), and lifestyle modifications such as improved glycemic control and alcohol/smoking cessation. While any obvious mechanical obstruction needs dilation, the role of empiric dilation is unclear. Although the complications of EIP are rare, it is essential to be aware of the possibility of esophageal stricture in the patients with EIP due to its potential for severe complications. These complications may include esophageal perforation, broncho-esophageal fistulas, pericardial and pleural effusions, or gastrointestinal bleeding [15]. Studies have shown a higher prevalence of EIP in the patients with esophageal carcinoma compared to those undergoing esophagography for other indications [16]. While no guidelines exist, surveillance endoscopy may be considered for EIP patients due to its association with esophageal malignancies.

## Conclusions

The association between HIV and esophageal intramural pseudodiverticulosis (EIP), especially when esophageal candidiasis is not present, is not frequently documented in the literature. However, it is possible that esophagitis in this patient population could contribute to the development of EIP. However, clinicians should be aware of the possibility of EIP in HIV patients who present with dysphagia. While esophageal candidiasis is a known risk factor for EIP, the absence of fungal elements in the presented case suggests that other factors such as that of esophagitis may contribute to the development of EIP in HIV patients. Considering EIP in the differential diagnosis of dysphagia in HIV patients can help ensure the timely recognition and appropriate management of this condition. Further research is needed to better understand the association between HIV and EIP, including the underlying mechanisms and risk factors involved.
